# Comparison of sennoside A + B and sodium picosulfate-based regimens for bowel preparation: a prospective randomized trial

**DOI:** 10.1007/s10151-026-03336-2

**Published:** 2026-05-14

**Authors:** İ. Durak, M. Özden, T. Düzenli, M. Sadeçolak, M. Kaya, H. Köseoğlu

**Affiliations:** 1 Department of Gastroenterology, Faculty of Medicine, Hitit University, Çorum, Turkey; 2 Department of Internal Medicine, Hitit University Çorum Training and Research Hospital, Çorum, Turkey; 3 Department of Gastroenterology, Faculty of Medicine, Samsun University, Çorum, Turkey

**Keywords:** Colonoscopy, Cathartics, Patient compliance, Health literacy

## Abstract

**Aim:**

Adequate bowel preparation is essential for high-quality colonoscopy and directly affects key quality indicators such as adenoma detection and cecal intubation. Although low-volume, nonpolyethylene glycol (PEG) regimens are widely used in clinical practice, real-world comparative data between stimulant-based preparations and sodium picosulfate-based combinations remain limited. This study aimed to compare the effectiveness and tolerability of sennoside A + B versus sodium picosulfate/magnesium oxide/citric acid (PM/Ca) using the Boston Bowel Preparation Scale (BBPS).

**Methods:**

This prospective, randomized, observer-blinded trial included adults aged 18–80 years undergoing elective outpatient colonoscopy. Participants were randomized 1:1 to receive either sennoside A + B oral solution (split-dose: 250 mL at 21:00 and 250 mL at 23:00) or PM/Ca (two sachets at 19:00 and 21:00), both administered with a low-residue diet and written instructions; rectal enemas were applied as part of the institutional protocol. Bowel cleanliness was assessed using BBPS. Adequate preparation was defined as BBPS > 6. Multivariable logistic regression was performed to identify independent predictors of adequate preparation.

**Results:**

A total of 705 patients were included in the primary analysis cohort (341 sennoside A + B; 364 PM/Ca). Adequate bowel preparation (BBPS > 6) was achieved more frequently in the sennoside A + B group than in the PM/Ca group (70.1% versus 56.3; *p* < 0.001), and the mean BBPS score was higher (6.64 ± 1.78 versus 5.93 ± 1.92; *p* < 0.001). Polyp detection rates were similar between groups (21.4% versus 26.6; *p* = 0.138). In multivariable analysis, higher education (OR 1.38; 95% CI 1.06–1.81; *p* = 0.016) and sennoside A + B (OR 1.77; 95% CI 1.29–2.43; *p* < 0.001) were independent predictors of adequate preparation, whereas age, BMI, sex, diabetes mellitus, and constipation were not. Preparation intolerance was more frequent in the sennoside A + B group (7.0% versus 0.7%; *p* < 0.001; tolerance analysis *n* = 824). Failure to reach the cecum did not differ significantly between regimens (9.3% versus 12.3%; *p* = 0.216; analyzed in the entire study population *n* = 791).

**Conclusions:**

In routine clinical practice, sennoside A + B provided higher cleansing efficacy than PM/Ca as reflected by higher BBPS scores and a greater probability of achieving adequate bowel preparation. However, PM/Ca demonstrated a more favorable tolerability profile. Bowel preparation selection should balance efficacy and tolerability while considering patient-related factors such as educational level.

*Trial registration:* ClinicalTrials.gov, NCT06580366.

## Introduction

Colonoscopy plays a central role in the evaluation of the colon and rectum, serving as an essential tool in colorectal cancer (CRC) screening programs as well as in the diagnostic assessment of a wide range of lower gastrointestinal conditions. It is commonly performed in patients presenting with symptoms such as anemia, altered bowel habits, rectal bleeding, or abdominal pain. Owing to its ability to directly visualize the colonic mucosa and allow for simultaneous therapeutic interventions, colonoscopy is widely regarded as the most comprehensive modality for the detection, diagnosis, and management of colorectal diseases, including both premalignant and malignant lesions [1–3].

The effectiveness of colonoscopy as a screening tool largely depends on procedural quality. Inadequate bowel preparation negatively affects cecal intubation rates, reduces adenoma detection rates, prolongs procedure time, and increases the risk of missed lesions, thereby compromising the effectiveness of CRC screening. Indeed, insufficient colonoscopy quality indicators have been shown to be associated with an increased risk of interval colorectal cancer. Therefore, adequate bowel preparation is widely recognized as an indispensable component of high-quality colonoscopy [4, 5].

Polyethylene glycol (PEG)-based regimens have long been used as the standard method for bowel preparation because of their well-documented efficacy and minimal impact on fluid and electrolyte balance [6]. However, PEG-based preparations require the ingestion of large volumes of fluid, which is frequently associated with nausea, vomiting, abdominal discomfort, and incomplete intake, ultimately leading to poor patient adherence and inadequate bowel cleansing in real-world practice. These limitations are particularly evident among elderly individuals, patients with comorbidities, and populations with lower health literacy [7].

In response to these challenges, current international guidelines recommend low-volume bowel preparation regimens as acceptable alternatives to high-volume PEG-based solutions [7]. Non-PEG preparations, including sodium picosulfate/magnesium citrate-based combinations and stimulant laxatives containing sennosides, are endorsed by guidelines for use in appropriately selected patients, provided that adequate bowel cleansing can be achieved. These agents exert their effects through different mechanisms, enhancing colonic motility and fluid secretion, while requiring substantially lower fluid volumes, which may improve patient tolerability and adherence in routine clinical practice [6].

Beyond clinical effectiveness and patient compliance, the economic implications of bowel preparation regimens have gained increasing importance in the context of CRC screening programs. Inadequate bowel preparation is associated with prolonged procedure times, reduced endoscopy unit efficiency, and the need for repeat colonoscopy, all of which contribute to increased healthcare resource utilization [8]. Consequently, bowel preparation strategies that improve cleansing adequacy and patient adherence may offer advantages not only from a clinical perspective but also in terms of healthcare system sustainability [8].

Although PEG alternatives are included in clinical guidelines and are widely used in daily practice, real-world data comparing the effectiveness of different non-PEG bowel preparation regimens using validated bowel cleanliness scales remain limited. In particular, direct comparisons between stimulant-based regimens and sodium picosulfate-based combinations with respect to adequate bowel preparation and colonoscopy quality outcomes are scarce. Therefore, this study aimed to compare the effectiveness and tolerability of sennoside A + B versus sodium picosulfate/magnesium oxide/citric acid (PM/Ca) using the Boston Bowel Preparation Scale (BBPS) in a routine clinical setting. Furthermore, we sought to identify independent predictors of adequate bowel cleansing to assist in individualizing preparation strategies.

## Methods

### Study design and registration

This study was designed as a prospective, randomized, observer-blinded interventional trial conducted at a tertiary care gastroenterology outpatient clinic. The study protocol was approved by the Hitit University Faculty of Medicine Ethical Committee (Approval No: 2024-43) and was registered in the ClinicalTrials.gov Protocol Registration and Results System (NCT06580366) prior to patient enrollment.

Importantly, the study was conducted in a real-world clinical setting without altering the routine workflow of the endoscopy unit. All bowel preparation regimens, patient instructions, timing of administration, and colonoscopy procedures were performed in accordance with standard daily clinical practice. No additional interventions, reminders, or protocol modifications beyond routine care were introduced for study purposes. This pragmatic approach was intentionally chosen to reflect the actual performance of bowel preparation regimens under everyday conditions and to enhance the external validity and generalizability of the findings.

### Study population

The study population consisted of adult patients aged 18–80 years who were scheduled for elective outpatient colonoscopy. All participants provided written informed consent before inclusion.

Inclusion criteria were adult patients scheduled for elective colonoscopy who were able to comply with bowel preparation instructions.

Exclusion criteria included a history of intra-abdominal surgery, chronic kidney failure, congestive heart failure, chronic liver failure, inflammatory bowel disease, known electrolyte imbalance, current hospitalization, known gastrointestinal motility disorders, known allergy or hypersensitivity to sennosid A + B or sodium picosulfate/magnesium oxide/citric acid (PM/Ca) components, and inability to complete the bowel preparation regimen.

**Fig. 1:**
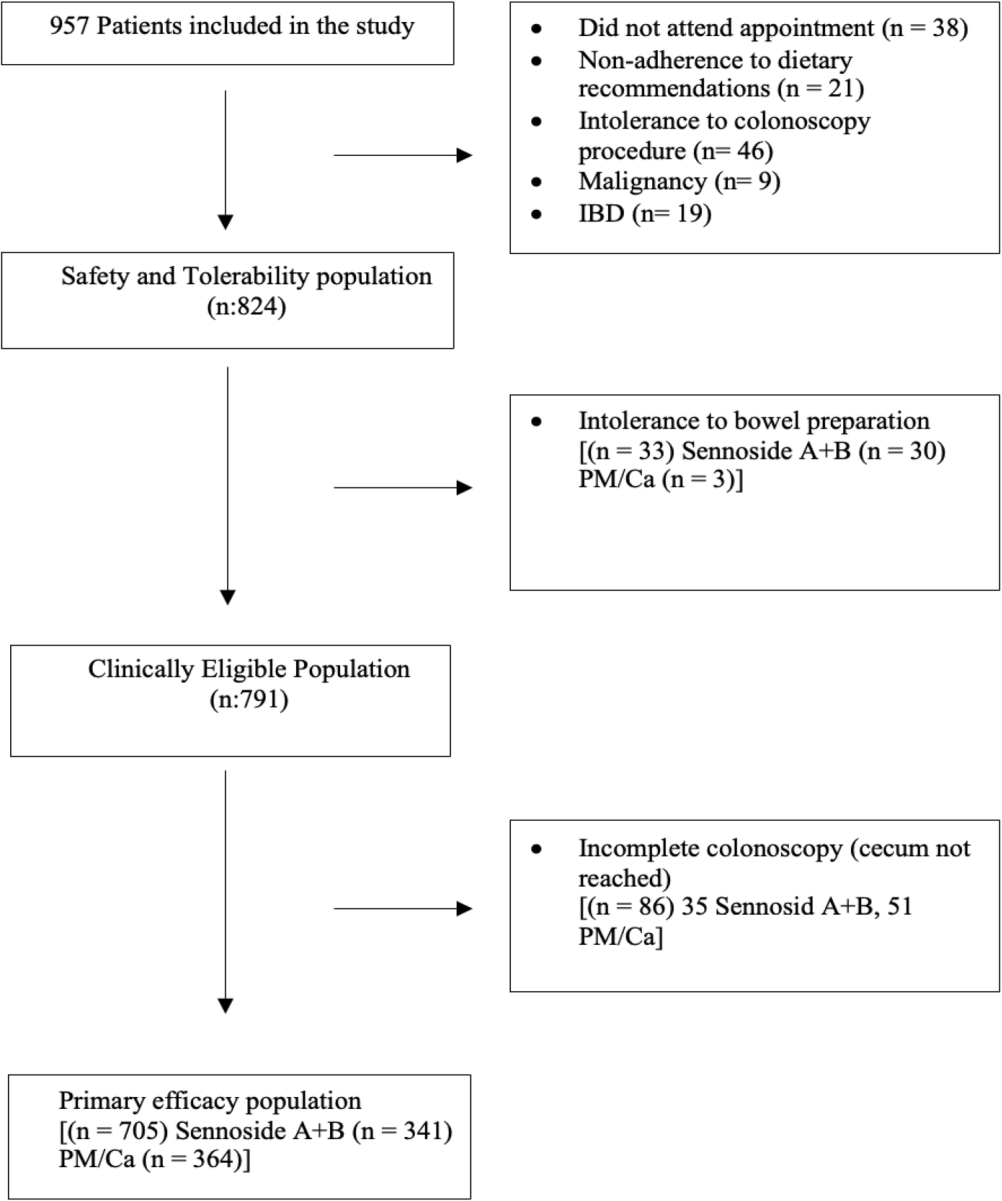
Flowchart of study

A total of 957 patients were assessed for eligibility. Of these, 133 patients were excluded due to failure to attend the appointment (*n* = 38), nonadherence to dietary recommendations (*n* = 21), intolerance to the colonoscopy procedure (*n* = 46), presence of malignancy (*n* = 9), or inflammatory bowel disease (*n* = 19). Consequently, 824 patients were included in the safety and tolerability population. Among these, 33 patients were excluded because of intolerance to bowel preparation, including 30 patients in the sennoside A + B group and 3 patients in the PM/Ca group, resulting in a clinically eligible population of 791 patients. Subsequently, 86 patients were excluded owing to incomplete colonoscopy, defined as failure to reach the cecum (35 in the sennoside A + B group and 51 in the PM/Ca group). Ultimately, 705 patients were included in the primary efficacy analysis, comprising 341 patients in the sennoside A + B group and 364 patients in the PM/Ca group (Fig. 1).

### Definitions

Body mass index (BMI) was calculated as weight in kilograms divided by the square of height in meters (kg/m^2^). Educational status was classified according to the highest level of formal education completed. High education was defined as completion of middle school, high school, or higher education, whereas low education referred to primary school education or less. Medication intolerance was defined as the inability to complete the prescribed bowel preparation regimen due to adverse effects such as nausea, vomiting, abdominal pain, bloating, or dizziness, resulting in incomplete intake or discontinuation of the preparation solution.

### Randomization and blinding

Eligible participants were randomly assigned in a 1:1 ratio to receive either sennosid A + B or sodium picosulfate/magnesium oxide/citric acid (PM/Ca) for bowel preparation. Randomization was performed using a computer-generated random sequence. Colonoscopy was performed by endoscopists who were blinded to the bowel preparation regimen, ensuring observer blinding during assessment of bowel cleanliness and procedural outcomes.

### Bowel preparation regimens

Participants received one of the following bowel preparation regimens, both commonly used in routine clinical practice. All participants were instructed to follow a low residue diet on the day preceding colonoscopy and to adhere strictly to written preparation instructions.

*Sennosid A + B regimen: *Sennosid A + B, a stimulant laxative derived from *Cassia senna*, was administered as an oral solution in a split-dose regimen. According to the product information, the solution contains 7.5 mg of sennosides A + B per 5 mL (1.5 mg/mL). Participants received 250 mL at 21:00 and an additional 250 mL at 23:00 on the evening before colonoscopy, in accordance with the manufacturer’s instructions.

*PM/Ca regimen:* The PM/Ca regimen consisted of sodium picosulfate (stimulant laxative) combined with magnesium oxide and citric acid (osmotic agents). Each sachet contained sodium picosulfate 10 mg, magnesium oxide 3.5 g, and anhydrous citric acid 10.97 g. Participants received two sachets in total, administered at 19:00 and 21:00 on the evening before colonoscopy, in accordance with the manufacturer’s instructions.

For both groups, the bowel preparation protocol, including medication use, timing, and recommended fluid intake, was explained in detail to all participants by the same healthcare professional using a standardized approach. In addition, the preparation instructions were provided in written form to each participant to ensure uniform understanding and adherence.

As part of the standard institutional bowel preparation protocol, a rectal enema containing sodium dihydrogen phosphate and disodium hydrogen phosphate (210 mL) was administered twice to all participants: once on the evening before colonoscopy and once on the morning of the procedure.

### Colonoscopy procedure

All colonoscopies were performed by experienced endoscopists using standard video colonoscopes. Cecal intubation was confirmed by visualization of the appendiceal orifice and ileocecal valve. Polyp detection and therapeutic interventions were performed in accordance with standard clinical practice.

### Assessment of bowel preparation quality

Bowel preparation quality was evaluated using the Boston Bowel Preparation Scale (BBPS), a validated scoring system assessing bowel cleanliness in three colonic segments (right, transverse, and left colon). Each segment was scored from 0 to 3, resulting in a total score ranging from 0 to 9, with higher scores indicating better bowel cleanliness.

Adequate bowel preparation was defined as a total BBPS score > 6 [9, 10].

### Outcome measures

The primary outcome was the effectiveness of bowel preparation, assessed using the Boston Bowel Preparation Scale.

Secondary outcomes included mean BBPS score, polyp detection rate, drug intolerance, and failure to reach the cecum.

### Statistical analysis

Statistical analyses were performed using IBM SPSS Statistics version 27 and RStudio for advanced modeling when required. The normality of continuous variables was assessed using the Kolmogorov–Smirnov test. Continuous variables with normal distribution were presented as mean ± standard deviation (SD), whereas non-normally distributed variables were expressed as median (interquartile range, IQR). Comparisons between groups were conducted using the independent samples *t*-test for parametric variables and the Mann–Whitney *U* test for nonparametric variables. Categorical variables were presented as number (percentage), and comparisons were performed using the Chi-square test when appropriate.

Correlations between continuous parameters were evaluated using Pearson correlation analysis. To identify independent predictors of adequate bowel preparation (defined as BBPS > 6), a multivariable logistic regression analysis was performed. Variables known to be clinically relevant or those with *p* < 0.10 in univariable analysis were included in the multivariable model. Results of the regression analysis were reported as odds ratios (ORs) with 95% confidence intervals (CIs). A two-tailed *p* value < 0.05 was considered statistically significant for all analyses.

### Ethical considerations

The study was conducted in accordance with the Declaration of Helsinki and approved by the Hitit University Faculty of Medicine Ethical Committee (Approval No: 2024-43). All participants provided written informed consent prior to participation.

## Results

Patient characteristics according to bowel preparation quality are presented in Table 1. Patients with inadequate bowel preparation (BBPS < 6) had a significantly higher prevalence of diabetes mellitus compared with those with adequate bowel preparation (42.9% versus. 34.7%, *p* = 0.034). In addition, the distribution of bowel preparation regimens differed significantly between groups, with PM/Ca being more frequently used among patients with inadequate bowel preparation (*p* < 0.001). Age, body mass index, sex, constipation, and education level did not differ significantly between patients with adequate and inadequate bowel preparation.

**Table 1:** Comparison of patients with adequate and inadequate bowel preparation according to BBPS cut-off (> 6)

Variable	Adequate BBPS (> 6) (*n* = 444)	Inadequate BBPS (< 6) (*n* = 261)	*p* value
Age (years)*	57.3 ± 13.3	58.0 ± 11.7	0.46
BMI (kg/m^2^) *	28.2 ± 5.3	28.9 ± 5.7	0.13
Male sex**	259 (58.3)	145 (55.6)	0.48
Diabetes mellitus**	154 (34.7)	112 (42.9)	**0.034**
Constipation**	38 (8.6)	24 (9.2)	0.773
Education level **			**0.026**
Low Education	271 (60.9)	183 (70.1)	
Higher education	173 (39.1)	78 (29.9)	
Bowel preparation regimen**			** < 0.001**
Sennosid A + B	239 (70.1)	102 (29.9)	
PM/Ca	205 (56.3)	159 (43.7)	

Bold highlights the statistical significance (for *P*< 0.05)

^*^t-test*,* mean ± SD; **Chi-Square, *n* (%)

*BMI* body mass index; *BBPS* Boston Bowel Preparation Scale; *PM/Ca* sodium picosulfate/magnesium oxide/citric acid

Baseline demographic and clinical characteristics according to bowel preparation regimen are summarized in Table 2. Age, body mass index, prevalence of diabetes mellitus, constipation, and education level did not differ significantly between patients receiving sennosid A + B and those receiving sodium PM/Ca.

**Table 2:** Comparison of baseline characteristics according to bowel preparation regimen (sennosid A + B versus PM/Ca)

	Sennosid A + B (*n* = 341, %48,4)	PM/Ca (*n* = 364, %51,6)	*P* score
Age*	58.17 ± 11.6	56.95 ± 12.0	0.203
BMI*	28.23 ± 4.6	28.70 ± 4.9	0.252
DM**	87 (25.5)	78 (21.4)	0.266
Constipation**	29 (8.5)	33 (9.1)	0.792
Education level**			0.127
Low Education	226 (66.3)	226 (62.1)	
Higher education	115 (33.7)	136 (37.9)	
BBPS (> 6) **	239 (70.1)	205 (56.3)	** < 0.001**
BBPS*	6.64 ± 1.78	5.93 ± 1.92	** < 0.001**
Right Colon	1.84 ± 0.7	1.62 ± 0.7	** < 0.001**
Transverse colon	2.21 ± 0.6	1.93 ± 0.6	** < 0.001**
Left colon	2.3 ± 0.6	2.08 ± 0.6	** < 0.001**
PDR**	73 (21.4%)	97 (26.6%)	0.138
Failure to reach the cecum^**,1^	35 (9,3)	51 (12,3)	0.216
Intolerance^**,2^	30 (7%)	3 (0.7%)	** < 0.001**

Bold highlights the statistical significance (for *P*< 0.05)

^*^t-test*,* mean ± SD; **Chi-Square, *n* (%)

^1^Failure to reach the cecum was analyzed in the clinically eligible population (*n* = 791*)*

^2^The tolerance analysis was assessed in the safety and tolerability population (*n* = 824)

*BMI* body mass index; *DM* diabetes mellitus; *BBPS* Boston Bowel Preparation Scale; *PM/Ca* sodium picosulfate/magnesium oxide/citric acid; *PDR* polyp detection rate

Adequate bowel preparation (BBPS > 6) was achieved significantly more frequently in the sennosid A + B group compared with the PM/Ca group (70.1% versus 56.3%, *p* < 0.001). Consistently, the mean BBPS score was higher among patients treated with sennosid A + B than those receiving PM/Ca (6.64 ± 1.78 versus 5.93 ± 1.92, *p* < 0.001). Segmental BBPS scores were also significantly higher in the sennosid A + B group for the right colon (1.84 ± 0.70 versus 1.62 ± 0.70, *p* < 0.001), transverse colon (2.21 ± 0.60 versus 1.93 ± 0.60, *p* < 0.001), and left colon (2.30 ± 0.60 versus 2.08 ± 0.60, *p* < 0.001). Polyp detection rates were comparable between the two regimens (21.4% versus 26.6%, *p* = 0.138). The rate of unsuccessful cecal intubation did not differ significantly between groups (9.3% versus 12.3%, *p* = 0.216). A total of 33 patients (4%) were unable to complete the bowel preparation regimen owing to medication intolerance [sennoside A + B: *n* = 30 (%7); PM/Ca: *n* = 3 (%0.7), *p* < 0.001)] and were excluded from the primary efficacy analysis.

The results of the multivariable logistic regression analysis evaluating independent predictors of adequate bowel preparation (BBPS > 6) are shown in Table 3. After adjustment for age, body mass index, sex, diabetes mellitus, constipation, education level, and bowel preparation regimen, higher education level remained independently associated with adequate bowel preparation (OR = 1.63, 95% CI 1.11–2.38; *p* = 0.016).

**Table 3:** Multivariable logistic regression analysis for BBPS > 6

Variable	OR	95% CI	*p* value
Age (per year)	1.00	0.99–1.02	0.561
BMI (kg/m^2^)	0.98	0.96–1.01	0.282
Male sex	0.76	0.54–1.07	0.117
Diabetes mellitus	0.72	0.50–1.03	0.078
Constipation	0.90	0.51–1.56	0.706
Higher Education	**1.63**	**1.11–2.38**	**0.012**
Senna preparation (versus PM/Ca)	**1.80**	**1.31–2.47**	** < 0.001**

Bold highlights the statistical significance (for *P*< 0.05)

Pseudo R^2^: 0.029; Likelihood ratio test: *p* = 0.0003

In addition, use of the sennosid A + B bowel preparation regimen was a strong independent predictor of adequate bowel cleansing compared with sodium picosulfate/magnesium oxide/citric acid (PM/Ca) (OR = 1.80, 95% CI 1.31–2.47; *p* < 0.001). Age, BMI, sex, diabetes mellitus, and constipation were not independently associated with achieving BBPS > 6.

**Fig. 2:**
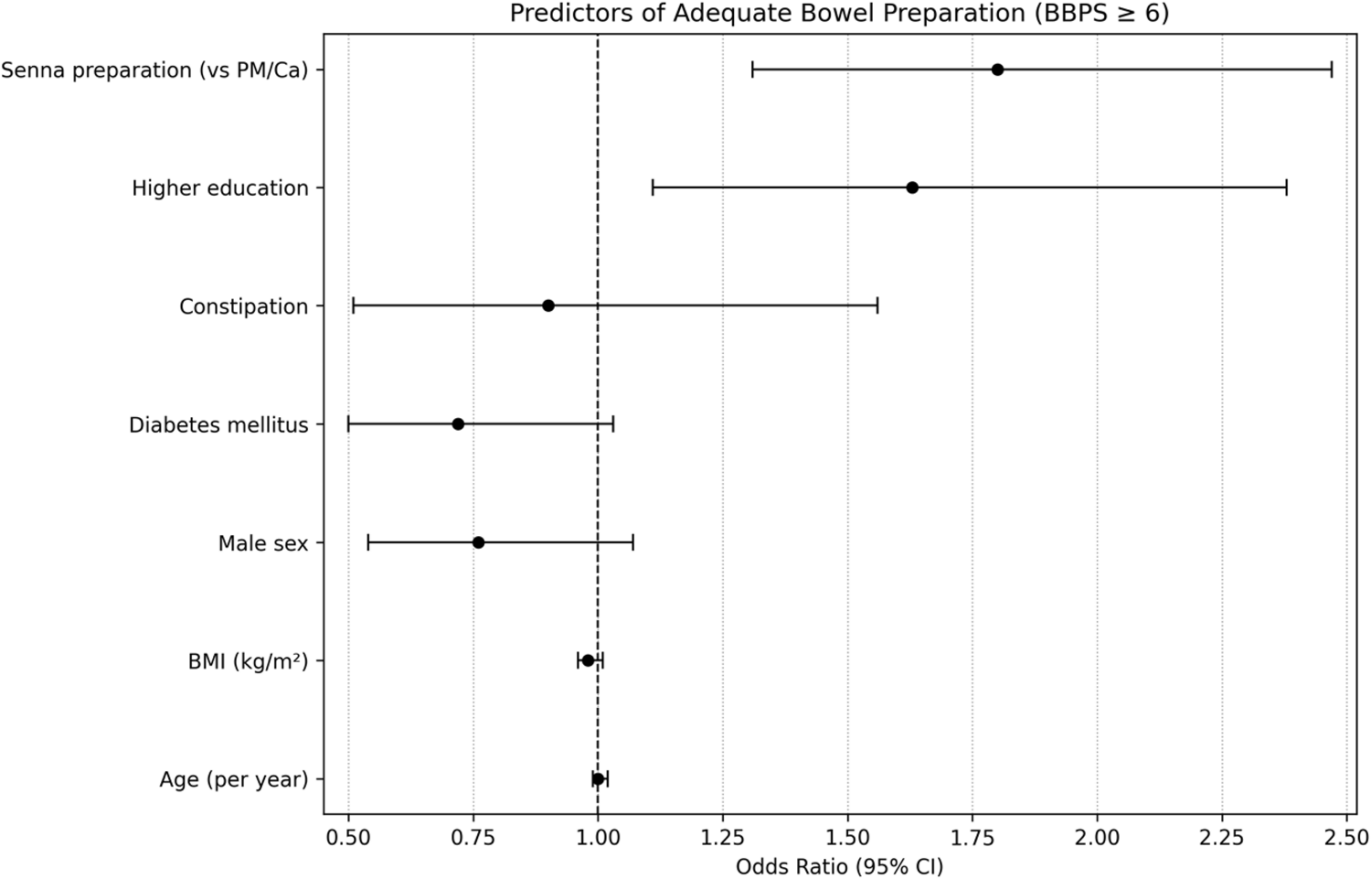
Predictors of adequate bowel preparation (BBPS > 6)

Figure 2 illustrates the adjusted odds ratios derived from the multivariable logistic regression model for predictors of adequate bowel preparation (BBPS > 6). Use of sennosid A + B was independently associated with higher odds of achieving adequate bowel preparation compared with PM/Ca (OR = 1.80, 95% CI 1.31–2.47). In addition, higher education level was also independently associated with increased odds of adequate bowel preparation (OR = 1.63, 95% CI 1.11–2.38). In contrast, age, body mass index, sex, diabetes mellitus, and constipation were not independently associated with achieving BBPS > 6, as their confidence intervals crossed unity.

Subgroup analysis of patients with unsuccessful cecal intubation according to bowel preparation regimen is presented in Table 4. Among patients in whom cecal intubation was unsuccessful, age, body mass index, sex, diabetes mellitus, constipation, and education level did not differ significantly between those receiving sennosid A + B and those receiving PM/Ca.

**Table 4:** Subgroup analysis of patients with unsuccessful cecal intubation according to bowel preparation method

	Sennosid A + B (*n* = 64)	PM/Ca (*n* = 38)	*p*
Age*	60.9 ± 12.4	57.2 ± 13	0.19
BMI*	28.7 ± 5,4	27.5 ± 5.5	0.31
Gender (Male)**	19 (54.3%)	22 (43.1%)	0.53
DM**	14 (40%)	17 (33.3%)	0.53
Constipation**	18 (51.4%)	27 (52.9%)	1.00
Educational Level**			0.34
< Low Education	37 (57.8%)	22 (57.9%)	
> High Education	27 (42.2%)	16 (42.1%)	

*t-test, Mean ± SD. **Chi-Square, *n* (%)

These findings indicate that, in this subgroup, patient-related demographic and clinical characteristics were comparable between bowel preparation regimens, suggesting that factors other than bowel preparation method may contribute to unsuccessful cecal intubation.

## Discussion

Ensuring optimal mucosal visualization during colonoscopy is a critical step that directly influences key quality indicators, including adenoma detection rates, procedure time, cecal intubation success, and the need for repeat examinations [11, 12]. Inadequate bowel preparation reduces the diagnostic value of colonoscopy and leads to increased costs and workload owing to unnecessary repeat procedures [13]. In the present study, the real-world effectiveness of two different bowel preparation regimens was compared. Our findings demonstrate that the sennoside A + B regimen achieved higher BBPS scores than PM/Ca in terms of cleansing efficacy, although it may be associated with certain disadvantages regarding tolerability. These results suggest that bowel preparation strategies should be individualized according to patient characteristics and clinical priorities before colonoscopy.

In our study, the rate of adequate bowel preparation was significantly higher in patients receiving sennoside A + B compared with those receiving PM/Ca (70.1% versus 56.3%), and the mean BBPS score was also significantly better in the sennoside A + B group. This association was independently confirmed in the multivariable regression analysis, demonstrating that the use of sennoside A + B increased the likelihood of achieving adequate bowel cleansing by approximately 77% compared with PM/Ca (OR = 1.77). In addition, a higher educational level was identified as an independent factor associated with a 38% increase in the probability of adequate bowel preparation (OR = 1.38). The effect of educational level is consistent with previous studies suggesting that patient adherence and the ability to understand preparation instructions play an important role in bowel cleansing quality [14]. These findings indicate that patient education and effective communication regarding bowel preparation protocols may directly contribute to improved clinical outcomes [15].

Although the adequate bowel preparation rate observed in the PM/Ca group was lower than that reported in some controlled clinical trials, this finding should be interpreted within the context of the real-world design of the present study [16]. Unlike highly standardized trial settings, our study reflects routine clinical practice, where variability in patient adherence and understanding of preparation instructions is unavoidable. In addition, a substantial proportion of the study population had a low educational level, which may disproportionately affect the effectiveness of low-volume, timing-sensitive regimens such as PM/Ca [17]. Consistent with this observation, educational level emerged as an independent predictor of adequate bowel preparation in our analysis. These findings suggest that the cleansing efficacy of PM/Ca may be more susceptible to patient-related and behavioral factors, particularly in populations with lower educational attainment, and highlight the importance of tailored patient education and regimen selection in daily practice.

The combination of sodium picosulfate, magnesium oxide, and citric acid, which is among the commonly used low-volume bowel preparation agents, has been reported in multiple studies to be well tolerated and easy to use. However, some comparative studies have suggested that the cleansing efficacy of PM/Ca may be lower than that of polyethylene glycol-based preparations and that BBPS scores may exhibit considerable variability [16, 18] Although sennoside A + B belongs to the class of stimulant laxatives, the available literature regarding its use for bowel preparation is limited, and studies directly comparing it with PM/Ca are scarce. From this perspective, the present study addresses an important knowledge gap and provides clinically relevant evidence by demonstrating superior cleansing efficacy of sennoside A + B compared with PM/Ca. Our findings are also consistent with other data suggesting that, with appropriate patient selection, low-volume and patient-friendly bowel preparation regimens can achieve adequate cleansing quality.

In the evaluation of bowel preparation regimens, tolerability and patient adherence are important determinants of clinical success in addition to cleansing efficacy [6]. Previous studies have reported that high-volume preparations may be poorly tolerated by some patients owing to their taste and volume [19]. In contrast, the PM/Ca combination, as a low-volume regimen, has been described as a favorable option in terms of tolerability in several studies, owing to better palatability and higher patient satisfaction [20].

In this context, differences in tolerability between the two regimens were additionally examined in our study, and patients who were unable to tolerate the preparation were evaluated at the subgroup level. This subgroup analysis suggested that the PM/Ca regimen exhibited a more favorable tolerability profile, whereas the sennoside A + B regimen demonstrated superior cleansing efficacy among patients who were able to complete the preparation and were included in the analysis. These findings are consistent with the existing literature indicating that low-volume preparations are generally better tolerated; however, they also suggest that an advantage in tolerability does not necessarily translate into higher cleansing efficacy. The higher cleansing performance observed with sennoside A + B in patients who tolerated the regimen underscores the importance of considering patient characteristics and tolerance profiles when selecting a bowel preparation strategy. Accordingly, in clinical practice, the choice of bowel preparation should be based on a balanced assessment of both efficacy and tolerability rather than on either parameter alone.

The overall rate of adequate bowel preparation observed in our cohort was relatively lower than that reported in highly controlled clinical trials, and this finding should be interpreted in the context of the study population and design. First, a substantial proportion of our patients had a low educational level, which has been consistently associated with poorer adherence to bowel preparation instructions and suboptimal cleansing quality [14]. Indeed, educational status emerged as an independent predictor of adequate bowel preparation in our multivariable analysis, supporting the critical role of health literacy in preparation success.

Second, unlike many previous studies conducted under tightly controlled experimental conditions, our study was performed in routine clinical practice, reflecting real-life patient behavior, variability in adherence, and practical limitations encountered in daily care. In real-world settings, factors such as incomplete intake, misunderstanding of instructions, and deviations from dietary recommendations are more common and may not be fully captured by standard exclusion criteria. Notably, similar findings have also been reported in other real-world randomized studies. In a multicenter trial comparing polyethylene glycol and sodium picosulfate/magnesium citrate, the median Ottawa Bowel Preparation Quality Scale score was 5 in both groups, indicating only moderate cleansing quality despite standardized protocols and trained endoscopists [16]. These data highlight the substantial heterogeneity of bowel cleansing in daily practice. Therefore, the relatively modest preparation rates observed in our study should not be interpreted as reduced efficacy of the regimens themselves, but rather as a realistic representation of bowel preparation performance in everyday clinical practice.

Polyethylene glycol-based regimens have long been regarded as the reference standard for bowel preparation owing to their proven cleansing efficacy and favorable safety profile, particularly in patients at risk for electrolyte imbalance. Current international guidelines continue to recommend PEG-based solutions as effective bowel preparation options. However, the large volume required for adequate cleansing has been consistently associated with reduced tolerability and lower patient adherence in real-world practice. In our clinical setting, PEG-based regimens are not routinely preferred, primarily because of these practical limitations rather than concerns regarding efficacy. These challenges may be further amplified in populations with limited health literacy, where completing high-volume and time-intensive preparation protocols can be difficult [7, 17, 21].

In addition to volume-related limitations, the economic burden of bowel preparation regimens represents an important consideration in daily practice [22]. In the present study, the total bowel preparation cost was approximately 12 USD in the sennoside A + B group and 10.5 USD in the PM/Ca group, including the cost of rectal enemas administered in both arms. These costs are substantially lower than the average cost of PEG-based regimens in our country, which is approximately 22 USD. From this perspective, lower-cost alternatives such as sennoside A + B and PM/Ca may offer pragmatic advantages in resource-limited settings by reducing preparation-related expenses without necessarily compromising cleansing efficacy in appropriately selected patients.

In our study, age, body mass index, diabetes mellitus, and constipation were not identified as independent predictors of adequate bowel preparation. The existing literature regarding the impact of these factors on bowel preparation quality is heterogeneous; while some studies have reported an association between advanced age, obesity, or diabetes and inadequate bowel preparation, others have failed to demonstrate a significant relationship [23–26]. This variability suggests that bowel preparation quality may be influenced not only by demographic and metabolic characteristics but also, and perhaps more importantly, by behavioral and procedural factors such as the type of preparation regimen, patient adherence, and the comprehensibility of preparation instructions. Indeed, in our study, the bowel preparation regimen and educational level emerged as independent predictors of adequate cleansing, indicating that pharmacological efficacy and patient adherence may play a more decisive role in preparation success. From this perspective, the presence of demographic risk factors alone does not appear sufficient to predict preparation failure, and patient-centered education and appropriate regimen selection seem to be of greater clinical importance.

In the subgroup analysis of patients in whom cecal intubation could not be achieved, no significant difference was observed between the bowel preparation regimens. However, in the present study, failure to reach the cecum was predominantly associated with insufficient bowel cleansing and markedly impaired visualization owing to residual fecal material. Although patients who reported nonadherence to preparation instructions or intolerance to the preparation were excluded by study design, the literature emphasizes that adherence issues such as incomplete intake of the preparation, inadequate or incorrect dietary restriction, and misinterpretation of preparation instructions may not always be recognized or reported by patients [7, 27]. Such unrecognized nonadherence can result in severely inadequate bowel preparation and consequently incomplete colonoscopy. Therefore, this small subgroup of patients in whom cecal intubation was not achieved should not be considered an appropriate population for comparing the true efficacy of bowel preparation regimens. Accordingly, the absence of a difference between the two regimens should be interpreted in the context of severely inadequate preparation, under which potential differences in regimen efficacy may be obscured.

Among the major strengths of this study are its randomized design and large sample size. The randomized comparison of sennoside A + B and PM/Ca bowel preparation regimens within the same clinical setting reduces potential selection bias and enhances the internal validity of the findings. The relatively large number of patients and the standardized implementation of preparation protocols further support the reliability of the results. In addition, bowel preparation quality was analyzed not only using the continuous BBPS score but also categorically based on the clinically relevant BBPS > 6 threshold, which improves the direct applicability of the findings to real-world practice. Collectively, these methodological features position our study as a robust comparative analysis with a strong methodological foundation.

Several limitations of this study should also be acknowledged. Although bowel preparation tolerability was assessed through intolerance-related exclusions and subgroup analyses, patient-reported outcomes such as satisfaction and detailed adverse event profiles were not systematically collected. Therefore, tolerability could be evaluated primarily in terms of the ability to complete the preparation rather than through comprehensive patient-reported measures. Furthermore, this was a single-center study, and variations in patient characteristics, endoscopy unit experience, and preparation protocols across different centers may limit the generalizability of the results. Nevertheless, the randomized design, large sample size, and standardized assessment methods remain key strengths that reinforce the internal validity of the study.

## Conclusions

In routine clinical practice, the sennoside A + B regimen provides superior cleansing efficacy compared with PM/Ca, as evidenced by higher BBPS scores and a greater likelihood of achieving adequate bowel preparation. This advantage is particularly pronounced among patients with higher education levels. However, PM/Ca remains a more favorable option regarding patient tolerability. Clinicians should balance these factors while considering patient-specific barriers, such as health literacy, when selecting the most appropriate bowel preparation strategy.

## Data Availability

The datasets generated and/or analyzed during the current study are available from the corresponding author on reasonable request.
